# Prolonged dual antiplatelet therapy in stable coronary disease: comparative observational study of benefits and harms in unselected versus trial populations

**DOI:** 10.1136/bmj.i3163

**Published:** 2016-06-22

**Authors:** A Timmis, E Rapsomaniki, S C Chung, M Pujades-Rodriguez, A Moayyeri, D Stogiannis, A D Shah, L Pasea, S Denaxas, C Emmas, H Hemingway

**Affiliations:** 1 The Farr Institute of Health Informatics Research, University College London, London, UK; 2 Barts and The London National Institute for Health Research, Cardiovascular Biomedical Research Unit, Bart’s Heart Centre, London, UK; 3 Department of Mathematics, University of Athens, Athens, Greece; 4 AstraZeneca, Luton, Bedfordshire, UK

## Abstract

**Objective** To estimate the potential magnitude in unselected patients of the benefits and harms of prolonged dual antiplatelet therapy after acute myocardial infarction seen in selected patients with high risk characteristics in trials.

**Design** Observational population based cohort study.

**Setting** PEGASUS-TIMI-54 trial population and CALIBER (ClinicAl research using LInked Bespoke studies and Electronic health Records).

**Participants** 7238 patients who survived a year or more after acute myocardial infarction.

**Interventions** Prolonged dual antiplatelet therapy after acute myocardial infarction.

**Main outcome measures** Recurrent acute myocardial infarction, stroke, or fatal cardiovascular disease. Fatal, severe, or intracranial bleeding.

**Results** 1676/7238 (23.1%) patients met trial inclusion and exclusion criteria (“target” population). Compared with the placebo arm in the trial population, in the target population the median age was 12 years higher, there were more women (48.6% *v* 24.3%), and there was a substantially higher cumulative three year risk of both the primary (benefit) trial endpoint of recurrent acute myocardial infarction, stroke, or fatal cardiovascular disease (18.8% (95% confidence interval 16.3% to 21.8%) *v* 9.04%) and the primary (harm) endpoint of fatal, severe, or intracranial bleeding (3.0% (2.0% to 4.4%) *v* 1.26% (TIMI major bleeding)). Application of intention to treat relative risks from the trial (ticagrelor 60 mg daily arm) to CALIBER’s target population showed an estimated 101 (95% confidence interval 87 to 117) ischaemic events prevented per 10 000 treated per year and an estimated 75 (50 to 110) excess fatal, severe, or intracranial bleeds caused per 10 000 patients treated per year. Generalisation from CALIBER’s target subgroup to all 7238 real world patients who were stable at least one year after acute myocardial infarction showed similar three year risks of ischaemic events (17.2%, 16.0% to 18.5%), with an estimated 92 (86 to 99) events prevented per 10 000 patients treated per year, and similar three year risks of bleeding events (2.3%, 1.8% to 2.9%), with an estimated 58 (45 to 73) events caused per 10 000 patients treated per year.

**Conclusions** This novel use of primary-secondary care linked electronic health records allows characterisation of “healthy trial participant” effects and confirms the potential absolute benefits and harms of dual antiplatelet therapy in representative patients a year or more after acute myocardial infarction.

## Introduction

National and international guidelines recommend the long term use of a range of treatments after acute myocardial infarction, but dual antiplatelet therapy with a P2Y_12_ receptor antagonist (clopidogrel, prasugrel, or ticagrelor) and aspirin is currently recommended for only up to one year.^1^ ^2^ ^3^ With increasing survival after the acute phase of myocardial infarction, however, there is a burgeoning population of stable patients requiring long term management for whom recent evidence from trials suggests that prolonging dual antiplatelet therapy beyond one year could provide continuing protection against cardiovascular events. The Prevention of Cardiovascular Events in Patients with Prior Heart Attack Using Ticagrelor Compared to Placebo on a Background of Aspirin (PEGASUS-TIMI-54) trial enrolled patients one to three years after an index acute myocardial infarction and showed that dual antiplatelet therapy with ticagrelor 60 mg compared with monotherapy with aspirin reduced the risk of cardiovascular death, myocardial infarction, or stroke by 16% but increased the risk of major bleeding by a factor of 2.4.^4^ Other studies on acute coronary syndrome have also reported point estimates for major adverse cardiac events favouring extended dual antiplatelet therapy compared with aspirin monotherapy; a contemporary meta-analysis reporting a 22% reduction in relative risk.^5^ These studies must be distinguished from those in percutaneous coronary intervention, in which dual antiplatelet therapy for less than a year seems effective for protecting against drug eluting stent thrombosis in most patients.^6^

It is not known how the balance of benefit and harm of extended dual antiplatelet therapy applies to the general population of unselected patients who survive the first year after acute myocardial infarction. Although it has been widely observed that event rates reported in trials tend to be lower than those reported in observational studies of hospital populations, patients in the PEGASUS-TIMI-54 trial were selected on “high risk” characteristics (age >65, renal impairment, two or more myocardial infarctions, and diabetes) to enhance the potential benefit of treatment. Registry outcome data for patients with acute coronary syndromes are often restricted to the first year.^7^ Patients who survive a year or more are an under-studied group, largely managed in primary care, for whom cardiovascular event rates have not been well defined. Population based electronic health records, such as those that exist in the United Kingdom, particularly when linked with disease, hospital admissions, and death registry data, provide a means of obtaining information on patient characteristics and outcomes that can then be compared with the more selected populations recruited within randomised controlled trials.^8^ ^9^ ^10^ ^11^ This approach has the potential to put into a public health context the interpretation of trial findings, but there have been few, if any, previous direct comparisons of trial risks with those from population based linked electronic health records.

We compared clinically representative populations with acute myocardial infarction drawn from CALIBER (ClinicAl research using LInked Bespoke studies and Electronic health Records) with the patients recruited into the PEGASUS-TIMI-54 trial. We examined patients’ characteristics, cardiovascular event rates, and rates of hospital admission for bleeding in the CALIBER populations meeting trial inclusion and exclusion criteria and compared these with patients participating in the PEGASUS-TIMI-54 trial to determine how this might affect clinical application of the results and the selection of patients for treatment with dual antiplatelet therapy a year or more after acute myocardial infarction.

## Methods

### Data sources

This observational cohort study was based on the CALIBER platform with linked electronic health records across primary care (the Clinical Practice Research Datalink (CPRD)), acute coronary syndrome registry (the Myocardial Ischaemia National Audit Project (MINAP)), hospital care (hospital episodes statistics), and cause specific mortality (Office for National Statistics).^12^ The CALIBER dataset comprises population based longitudinal patient data from linked electronic health records. The patients included have been shown to be representative of the whole population of England in terms of age, sex, ethnicity, and overall mortality,^13^ ^14^ ^15^ and we have extensively validated cardiovascular risk factors, including age, sex, deprivation, smoking, blood pressure, and type 2 diabetes, against a wide range of cardiovascular diseases.^16^ ^17^ ^18^ ^19^ ^20^ CALIBER contains primary care data from CPRD, with information on anthropometric measurements, laboratory tests, clinical diagnoses (coded with the Read clinical coding system), prescriptions (recorded with Multilex), and medical procedures. MINAP is a national registry of patients admitted to hospital with acute coronary syndromes. Hospital episode statistics provide information on diagnoses (coded with ICD-10 (international classification of diseases, 10th revision)) and medical procedures (coded with the Office of Population Censuses and Surveys—Classification of Interventions and Procedures, version 4 (OPCS-4)) related to all elective and emergency hospital admissions across all NHS (National Health Service) hospitals in England.

### Study populations

The CALIBER population was selected from patients admitted to hospital with a primary diagnosis of acute coronary syndrome (acute myocardial infarction or unstable angina) from April 2005 to March 2010 (fig 1), before ticagrelor was in use in the UK. A “real world” population of patients who were stable after acute myocardial infarction was identified, comprising 7328 patients who survived at least a year after the index admission, remained registered with the participating general practice, and had no further admissions for acute myocardial infarction during the year. From this real world population, we identified two further CALIBER populations. The first was a “high risk” population of 5279 patients who met the inclusion criteria for PEGASUS-TIMI-54 (age ≥65, or ≥50 with diabetes, renal disease, or a second previous myocardial infarction). We were unable to select on the basis of multivessel coronary disease, which was not coded in CALIBER. The second population comprised a “target population” of 1676 patients eligible for PEGASUS-TIMI-54 who met the trial’s high risk inclusion criteria (see above) and also exclusion criteria (listed against 3603 patients, fig 1). We set the study entry date (stable myocardial infarction date) to 12 months after the date of index acute myocardial infarction. Patients were followed up until death, transfer out of the general practice, or end of the study period (24 March 2010). Patients’ characteristics in the three CALIBER populations were compared with each other and with the trial participants.

**Figure f1:**
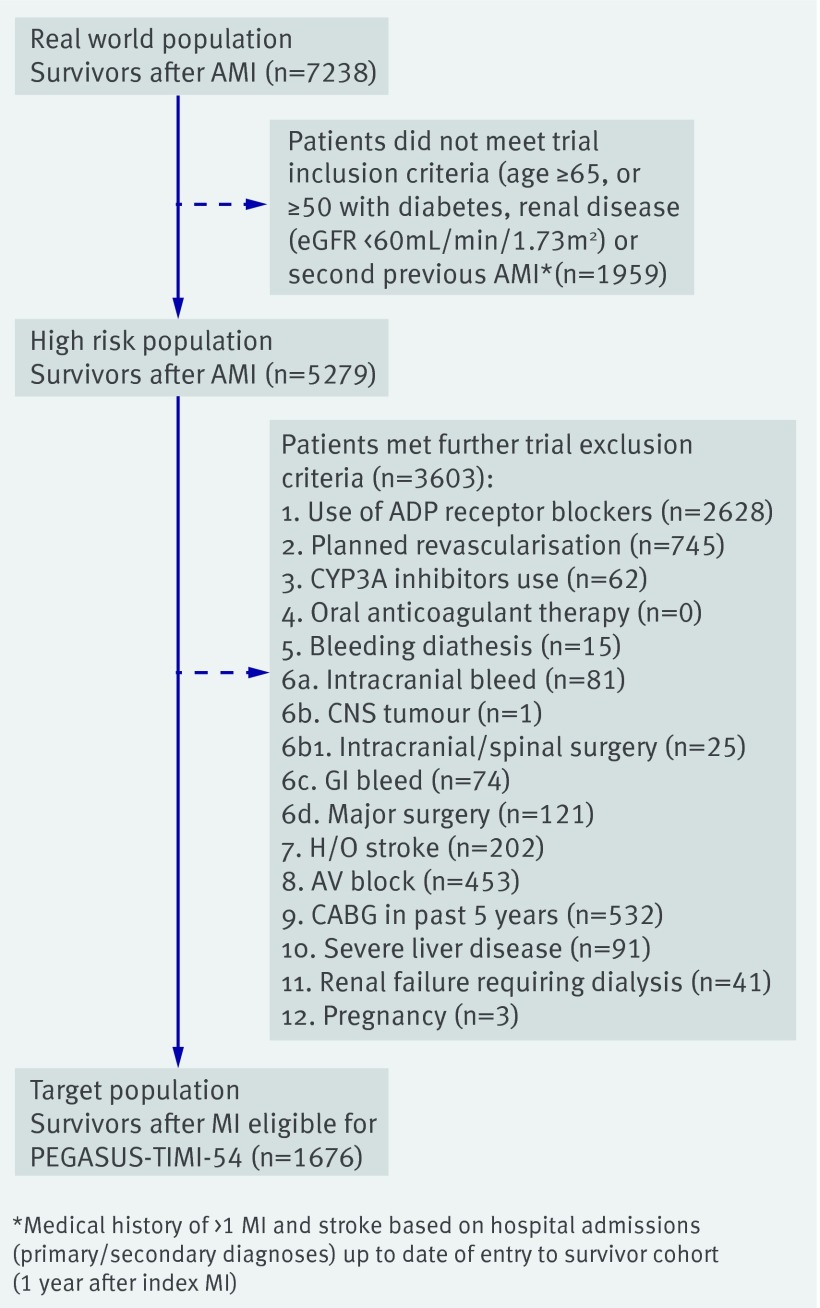
**Fig 1** Selection of participants who survived one year or more after myocardial infarction (MI) from CALIBER, April 2005-March 2010

### Baseline characteristics

We recorded age, sex, body mass index (BMI), smoking status (current, former, never), diabetes (use of insulin, metformin, or sulphonamides or a diagnosis recorded any time before study entry), diagnosis of hypertension, percutaneous coronary intervention or coronary artery bypass grafting procedures performed in the 12 month period between the index event and study entry date, and medical history (more than one hospital admission for acute myocardial infarction, renal disease, heart failure, peripheral arterial disease, atrial fibrillation, stroke, hospital admission for bleeding, chronic obstructive pulmonary disease, cancer, and dementia (diagnostic codes or drugs for treatment)), as recorded in primary or secondary care before the study entry date. Moderate/severe renal disease was defined on the basis of diagnoses in secondary care. ST elevation myocardial infarction (STEMI)/non-STEMI was determined from MINAP in 80% of cases but was unspecified in the remainder for whom the type of acute myocardial infarction was imputed based on ICD-10 code for hospital admission.^21^ Before imputation we tested the method in patients with documented acute myocardial infarction phenotype, showing agreement in 86% of cases. Diagnosis ICD-10 codes and phenotyping algorithms are available via the https://www.caliberresearch.org/portal online resource.

### Endpoints

The cardiovascular endpoint, matching the primary efficacy endpoint in the trial, was a composite of acute myocardial infarction, stroke, or death from cardiovascular disease. The bleeding endpoint, a proxy for the primary safety endpoint of TIMI major bleeding in the trial, was “fatal, severe, or intracranial bleeding” defined as a composite of fatal bleeding, bleeding requiring transfusion, hospital admission for bleeding with length of stay seven days or more, or intracranial bleeding (ICD-10 codes shown in table A in appendix 1). We also analysed fatal or intracranial bleeding, defined as bleeding recorded as the primary cause of death, hospital admission for bleeding within seven days before death, or hospital admission for intracranial bleeding (appendix 1).

### Patterns of drug treatment for cardiovascular disease

We examined patterns in drug use for secondary prevention of cardiovascular disease, including statins, aspirin, ADP inhibitor antiplatelet agents (mostly clopidogrel), dual antiplatelet therapy (aspirin combined with another antiplatelet agent), β blockers, angiotensin converting enzyme inhibitors (ACEIs), angiotensin receptor blockers (ARBs), oral anticoagulants (99% warfarin), proton pump inhibitors, and antidiabetic drugs. British National Formulary (BNF) codes for drugs are available in table B in appendix 1. Beginning from the index admission for acute myocardial infarction and for the first, second, and third years of follow-up since the date patients were stable after myocardial infarction (a year after acute index admission), we assessed the prescription continuation rates (period between the first and last prescription date assuming complete adherence within this interval) adjusted for censoring (cardiovascular event, death, or lost to follow-up) by the Kaplan-Meier method. Prescription use at baseline was classified as current/recent if the most recent prescription was active at the index date or ended within the 30 days before the index date.

### Statistical analysis

For each endpoint we estimated the incidence rate (total number of events observed divided by the total number of person years of observation), events per patient year rates during follow-up, and by specific time periods (0-1, 2-3, >3 years during period of stability) in each patient population. We applied the intention to treat relative risks from the trial (ticagrelor 60 mg data) to CALIBER study populations to estimate the number of ischaemic events prevented or fatal, severe, or intracranial bleeds caused per 10 000 treated per year. We compared observed risks between the different study populations by estimating the cumulative risks and 95% confidence intervals over the first three years of follow-up. In the population after myocardial infarction we evaluated the prognostic ability of clinical characteristics with respect to ischaemic outcome based on risks from the Cox proportional hazards model adjusted for age, sex, history of diabetes, more than one previous acute myocardial infarction, and eGFR <60 mL/min/1.73m^2^. Data were complete for all adjustment factors except eGFR, which was 86% complete, and hazard ratios were therefore estimated in complete cases analyses. We verified the proportional hazards assumption of the Cox model by plotting the Schoenfield residuals. All analyses were performed with the statistical package R (version 15.2), SAS (version 9.3) and STATA (version 13.1).

### Bias

Our real world patients were sourced from linked electronic health records, without exclusion and with >96% successful matching across CALIBER’s four data sources.^11^ ^12^ Biases in recruitment and follow-up were thereby minimised while the ascertainment of cardiovascular outcomes was enhanced.

### Patient involvement

No patients were involved in setting the research question or the outcome measures, nor were they involved in developing plans for design or implementation of the study. No patients were asked to advise on interpretation or writing up of results. There are no plans to disseminate the results of the research to study participants or the relevant patient community. 

## Results

The “real world” population for our analysis comprised 7238 patients recorded in CALIBER with acute myocardial infarction who survived for a year or more without another infarction (fig 1). From this population we identified a “high risk” population of 5279 (72.9%) patients who met the PEGASUS-TIMI-54 trial inclusion criteria and a “target” population of 1676 (23.1%) patients who met both the inclusion and exclusion criteria for the trial (table 1). Follow-up, starting 12 months after the index event, was for a median of 1.5 (interquartile range 0.7-2.5) years.

**Table 1 tbl1:** Baseline characteristics of populations of patients who survived acute myocardial infarction (AMI) defined in this study and equivalent characteristics from PEGASUS-TIMI-54 trial. Figures are numbers (percentage) unless stated otherwise

	CALIBER observational data from population of England			PEGASUS-TIMI-54 placebo group (n=7067)
	All post-MI survivors “real world” (n=7328)	Met trial inclusion criteria “high risk” (n=5279)	Met trial inclusion and exclusion criteria “target” (n=1676)	
Index myocardial infarction:				
STEMI	2827 (39.1)	1809 (34.3)	599 (35.7)	3809 (54.0)
Non-STEMI	4411 (60.9)	3470 (65.7)	1077 (64.3)	2843 (40.3)
Median (IQR) years after index AMI	1.0 (1-1)	1.0 (1-1)	1.0 (1-1)	1.7 (1.2-1.3)
Baseline characteristics:				
Mean (SD) age (years)	69.6 (13.0)	75.3 (9.5)	77.0 (9.6)	65.4 (8.3)
Women	2461 (34.0)	2088 (39.6)	814 (48.6)	1717 (24.3)
White	5924 (94.4)	4174 (94.2)	1350 (95.7)	6124 (86.7)
Mean (SD) weight (kg)*	79.6 (17.5)	77.2 (16.8)	74.8 (17.4)	81.8 (16.6)
Hypertension	4543 (62.8)	3677 (69.7)	1090 (65.0)	5484 (77.6)
Hypercholesterolemia	NA	NA	NA	5451 (77.1)
Current smoker*	1065 (15.7)	572 (11.6)	162 (10.6)	1143 (16.2)
Diabetes mellitus	1522 (21.0)	1449 (27.5)	392 (24.4)	2257 (31.9)
Multivessel coronary artery disease	NA	NA	NA	4213 (59.6)
History of PCI†	3025 (41.8)	1840 (34.9)	391 (23.3)	5837/7066 (82.6)
More than one myocardial infarction	830 (11.5)	804 (15.2)	191 (11.4)	1188 (16.8)
Peripheral arterial disease	449 (6.2)	400 (7.6)	101 (6.0)	404 (5.7)
eGFR <60 mL/min/1.73m^2^*	2402 (37.5)	2383 (49.9)	797 (52.3)	1649 (23.6)
Drug treatment:				
Any aspirin	5726 (79.1)	4118 (78.0)	1250 (74.6)	7057 (99.9)
Clopidogrel	3713 (51.3)	2628 (49.8)	0 (0)	NA
Statins	6262 (86.5)	4500 (85.2)	1335 (79.7)	6583 (93.2)
β blockers	5098 (70.4)	3574 (67.7)	1065 (63.5)	5878 (83.2)
ARBs/ACEIs	5793 (80.0)	4145 (78.5)	1283 (76.6)	5697 (80.6)

STEMI=ST elevation myocardial infarction; IQR=interquartile range; NA=not available; PCI=percutaneous coronary intervention; eGFR=estimated glomerular filtration rate; ARBs=angiotensin II receptor blockers; ACEIs=angiotensin converting enzyme inhibitors.

*Information from CALIBER available for 65% for weight, 94% for smoking status, and 88% for eGFR.

†In past 365 days.

### Population characteristics

The CALIBER target population was older (median age 12 years higher) and included proportionately more women and more patients with non-STEMI compared with the PEGASUS-TIMI-54 trial population (table 1). Differences were similar for CALIBER’s high risk population and were only slightly less for CALIBER’s real world population. Analysis of the high risk inclusion criteria for the trial showed that age ≥65 characterised 90%, 89%, and 64% of CALIBER’s target, high risk, and real world populations, respectively, compared with 55% of the PEGASUS-TIMI-54 trial population. Renal dysfunction (eGFR <60 mL/min/1.73m^2^) was also more prevalent in the CALIBER populations, but other risk factors, including second previous acute myocardial infarction and diabetes, were less prevalent compared with the trial. Baseline use of aspirin, β blockers, and statins was lower in the three CALIBER populations compared with the trial (table 1), and for each CALIBER population there were parallel declines in use during follow-up (fig 2, table C in appendix 1). This was most marked for ADP receptor inhibitors, which declined to <10% at three years.

**Figure f2:**
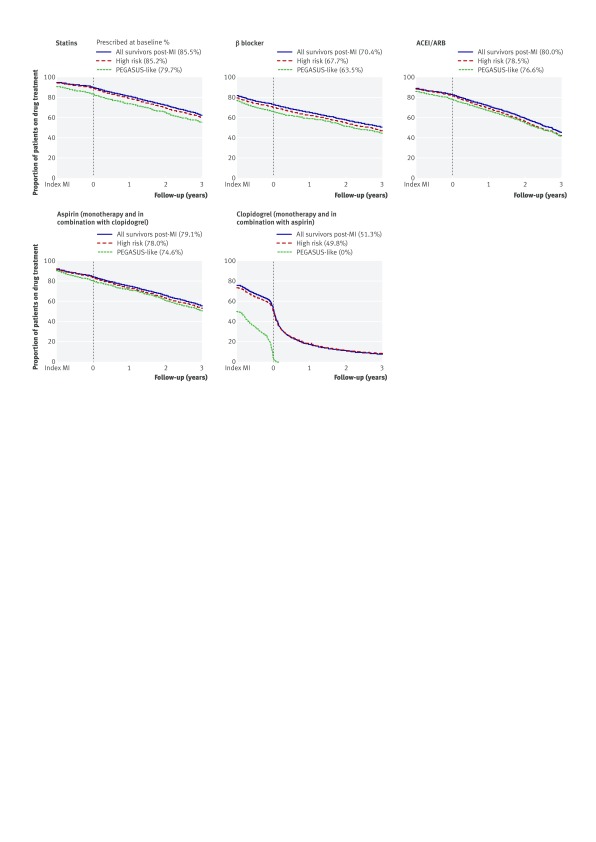
**Fig 2** Proportion of patients prescribed drugs for secondary prevention from date of index MI (one year before study entry) and up to three years of follow-up adjusted for censoring (assuming continuous coverage between first and last prescriptions issued). ACEI=angiotensin converting enzyme inhibitors; ADP=adenosine diphosphate; ARBs=angiotensin II receptor blockers

### Cardiovascular events

In CALIBER’s target, high risk, and real world populations the three year Kaplan-Meier rates for a composite outcome of acute myocardial infarction, stroke, or fatal cardiovascular disease were 18.8% (95% confidence interval 16.3% to 21.8%), 21.7% (20.1% to 23.3%), and 17.2% (16.0% to 18.5%), respectively, compared with 9.04% in the trial placebo group (table 2, fig 3). Patterns of all cause mortality were similar (fig A in appendix 2). With adjustment for age and sex the three year Kaplan-Meier composite outcome rate for the target population declined (13.9%, 10.8% to 17.9%), and the difference between the high risk and real world populations largely disappeared (17.9% (15.9% to 0.2%) and 17.2% (15.5% to 19.1%)). In CALIBER subgroups defined by age ≥65, one or more previous acute myocardial infarctions, diabetes, and renal disease, the age-sex adjusted risk of recurrent myocardial infarction, stroke, or death was 24.8%, 32.3%, 20.8%, and 22.8%, respectively (fig 4). Applying three year relative risk reduction for trial participants treated with ticagrelor 60 mg daily, we calculated that 101 (95% confidence interval 87 to 117) ischaemic events were prevented per 10 000 treated per year for the target group. The same relative risk reduction applied to the high risk and real world populations suggested at least 116 (108 to 125) and 92 (86 to 99) events were prevented.

**Table 2 tbl2:** Observed cumulative event rate (%) of clinical outcomes and number of events prevented or harms caused, applying trial results to UK patients who survived myocardial infarction (MI) defined in study (with 95% CI)

	Population based observational data			PEGASUS-TIMI-54 trial placebo arm (n=7067)
	All post-MI survivors “real world” (n=7238)	Met trial inclusion criteria “high risk” (n=5279)	Met trial inclusion and exclusion criteria “target” (n=1676)	
**MI/stroke/fatal cardiovascular disease**				
3 year cumulative risk (%)	17.2 (16.0 to 18.5)	21.7 (20.1 to 23.3)	18.8 (16.3 to 21.8)	9.04
No of events prevented/year/per 10 000 patients treated applying risk reduction in PEGASUS-TIMI-54 trial*	92 (86 to 99)	116 (108 to 125)	101 (87 to 117)	—
**Fatal, severe, or intracranial bleeding**				
3 year cumulative risk (%)	2.3 (1.8 to 2.9)	3.0 (2.4 to 3.8)	3.0 (2.0 to 4.4)	1.26
No of excess harms/year/10 000 patients treated applying risk increase in PEGASUS-TIMI-54 trial*	58 (45 to 73)	75 (60 to 95)	75 (50 to 110)	—

*PEGASUS-TIMI-54 trial estimates (ticagrelor 60 mg *v* placebo; intention to treat estimates) for cardiovascular death, stroke, or myocardial infarction (hazard ratio 0.84, main report^4^), TIMI major bleeding (relative risk 1.75, supplementary appendix in main report^4^) were used to calculate cardiovascular events prevented and harms caused per 10 000 treated per year.

**Figure f3:**
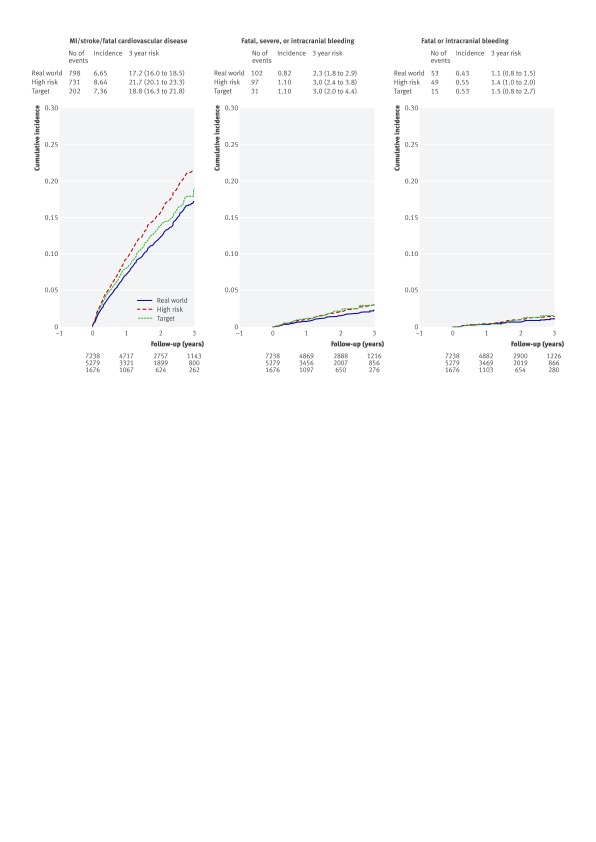
**Fig 3** Kaplan-Meier risks of ischaemic and bleeding events in CALIBER’s real world, high risk, and target populations of patients surviving one year or more after acute myocardial infarction

**Figure f4:**
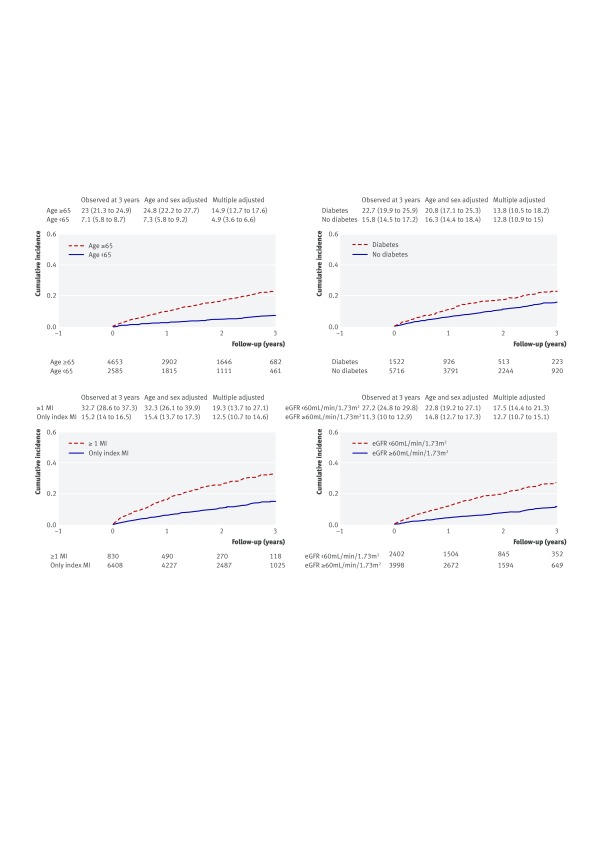
**Fig 4** Kaplan-Meier risks of myocardial infarction/stroke/fatal cardiovascular disease for patient subgroups defined by age, diabetes, one or more previous acute MI, and renal disease (indicated by eGFR) in CALIBER “real world” population. Age adjusted except for age subgroup analyses. Multivariate adjustment included age (not for age subgroup analyses), sex, diabetes, more than one MI, and renal disease. Reported CVD risk at third year of follow-up for reference patient characteristics: age 71 (median age of study population), male, no diabetes, no more than one previous MI, and no chronic renal disease

### Bleeding events

In CALIBER’s target, high risk, and real world populations the three year Kaplan-Meier rates for fatal, severe, or intracranial bleeding were 3.0% (95% confidence interval 2.0% to 4.4%), 3.0% (2.4% to 3.8%), and 2.3% (1.8% to 2.9%), respectively, compared with 1.26% for TIMI major bleeding in the trial placebo group (table 2, fig 3). The three year rates of fatal or intracranial bleeding in the three CALIBER populations were about twice the 0.6% rate in the trial. Applying three year relative risk increase for trial participants treated with ticagrelor 60 mg daily, we calculated that an extra 75 (95% confidence interval 50 to 110), 75 (60 to 95), and 58 (45 to 73) fatal, severe, or intracranial bleeds per 10 000 treated per year occurred in the target, high risk, and real world populations, respectively. For fatal or intracranial bleeding, excess events per 10 000 treated per year were 10 (6 to 18), 10 (7 to 14), and 8 (6 to 10), respectively (table D in appendix 1).

### Sensitivity analysis

We found high event rates among patients censored in the first year, which did not differ according to clopidogrel prescription status at study entry. Event rates also did not differ according to status two years after the index acute myocardial infarction (approximating to the median 1.7 years that characterised PEGASUS-TIMI-54 trial entry), suggesting that the observed difference in event rates between CALIBER and PEGASUS-TIMI-54 is unlikely to be biased by continuing clopidogrel use (table E in appendix 1).

## Discussion

### Principal findings

Both trial evidence and real world evidence are required to inform decisions about adding prolonged dual antiplatelet therapy to the combination of drugs recommended for secondary prevention of myocardial infarction. In this novel comparison of participants in a major randomised trial (PEGASUS-TIMI-54) with unselected population based data from linked primary and secondary care electronic health records (CALIBER)^4^ ^11^ we found that only about a quarter of all patients a year after acute myocardial infarction met trial inclusion and exclusion criteria. The cardiovascular risk in this “target” population was high compared with the trial population, with about twice the rate of non-fatal and fatal cardiovascular events in the subsequent three years. Our analysis indicates that addition of ticagrelor to the treatment regimen of these patients might prevent 101 of these events each year for every 10 000 patients treated, at the cost of 75 major bleeds. This ratio is almost identical to the calculation reported in the main trial results (42 *v* 31), although the absolute magnitude of potential net benefit of 26 is greater in the real world data.

### Comparison with previous studies

In comparing the population characteristics and outcomes of PEGASUS-TIMI-54 with real world populations drawn from electronic health records we have contextualised the trial’s findings and potential relevance to patient care. Previous studies have reported the limited generalisability of clinical trial populations, but comparisons have usually been with small hospital based samples without development of target populations defined by inclusion and exclusion criteria to allow more precise assessment of patient representativeness.^22^ ^23^ ^24^ These studies have been further limited by their focus on patients’ characteristics without consideration of outcomes to allow estimation of how treatment effects might translate into benefits for real world patients. We dealt with these limitations by providing an electronic health record resource to develop a target population drawn from primary care with follow-up for benefit and harm outcomes.

### Mismatch between target and trial populations

The novel use of primary-secondary care linked electronic health records has shown a large “healthy trial participant” effect, and, despite only about a quarter of our real world patients meeting PEGASUS-TIMI-54 inclusion and exclusion criteria, reflecting the limited generalisability of trial populations, their cardiovascular outcomes were considerably worse. The mismatch between the target and trial populations was reflected in differences in demographic and clinical characteristics, the target population being considerably older and with a higher proportion of women. The preponderance of non-STEMI in the target population is consistent with many recent reports^25^ ^26^ ^27^ and, together with the earlier recruitment start date compared with the trial population (2005 *v* 2010), accounts for the relatively low rate of percutaneous coronary intervention that we and others have previously reported for UK hospitals.^28^ Many real world patients who were receiving dual antiplatelet therapy for a year or more year after acute myocardial infarction were excluded in development of the target population, and this might have resulted in minor exaggeration of healthy trial participation as the trial patients were enrolled during a wider, one to three year recruitment window. Nevertheless, our comparison of patients’ characteristics in the CALIBER and PEGASUS-TIMI-54 populations was revealing in helping to understand the differences in cardiovascular outcome and the potential generalisability of the trial results. The methods we report are scalable, and our study suggests that such linked electronic health record resources could provide useful “real world evidence” for trials in other clinical settings.

### Cardiovascular risk late after myocardial infarction

A major finding in our study was the high cardiovascular risk of the whole population of patients who survived a year or more after acute myocardial infarction, consistent with a report from Sweden^29^ published before the PEGASUS trial results were available. Thus, the patients in CALIBER’s target population had a three year event rate twice that of participants in the trial, no doubt reflecting differences in simple risk factors,^30^ ^31^ ^32^ with adjustment for age and sex resulting in substantial reductions in event rates. The high event rate in CALIBER’s target population showed only minor attenuation in the real world population, indicating comparable potential for risk reduction with dual antiplatelet therapy given to all patients surviving a year or more after acute myocardial infarction. Accordingly, our estimation of ischaemic events prevented by ticagrelor 60 mg was similar for both target and real world populations and yet was greater for the high risk population defined by trial inclusion criteria. The Committee for Medicinal Products for Human Use of the European Medicines Agency has recently claimed that in patients with previous acute myocardial infarction who are at high risk of atherothrombotic events, treatment with ticagrelor 60 mg can be started as continuation treatment after an initial one year of treatment with dual antiplatelet therapy.^33^ Our findings support this view and indicate that the potential for dual antiplatelet therapy for a year or more after acute myocardial infarction to deliver clinical benefit is likely to be greatest in high risk subgroups, such as those defined in figure 4, that fulfil PEGASUS-TIMI-54 inclusion and exclusion criteria

### Benefits and harms of prolonged dual antiplatelet therapy

Only about half of CALIBER’s populations were prescribed clopidogrel at baseline (a year or more year after acute myocardial infarction), and prescription rates fell sharply thereafter in accordance with current guideline recommendations. Nevertheless, a small proportion of patients in CALIBER’s high risk and real world populations remained taking clopidogrel well after the first year (table C in appendix 1), but the analysis in table E in appendix 1 shows that outcomes were similar regardless of ongoing treatment, indicating that observed differences in event rates between CALIBER and PEGASUS-TIMI-54 were unlikely to be biased by continual clopidogrel use. The results of PEGASUS-TIMI-54 suggest that stopping P2Y_12_ receptor antagonists after the first year might be a missed treatment opportunity for reducing ischaemic events. The potential benefit of adding, or continuing, a P2Y_12_ receptor antagonist, however, must be weighed against the increased risk of bleeding reported in the trial. Fatal, severe, or intracranial bleeding, an approximation of the TIMI major bleeding endpoint in PEGASUS-TIMI-54, occurred more commonly in CALIBER’s target population than in the trial population, but our estimation of bleeding events caused by ticagrelor in the real world population was 25% lower than cardiovascular events prevented. In selecting patients for dual antiplatelet therapy for a year or more after acute myocardial infarction, the balance between cardiovascular benefit and risk of bleeding needs careful consideration, with recognition that patients and clinicians might have different views about their relative importance. The cumulative three year incidence of the most feared bleeding endpoints— fatal or intracranial haemorrhage—was only 1.4% in the high risk population for whom the benefits of treatment in terms of cardiovascular events prevented was greatest. Nevertheless, this residual risk of bleeding emphasises the importance of PEGASUS-TIMI-54 exclusion criteria, particularly previous stroke or recent anticoagulation therapy, in selecting patients for dual antiplatelet therapy.

Our analyses of benefit and harm suggested a net benefit of treatment with dual antiplatelet therapy for a year or more after acute myocardial infarction, reflected in the 99 target population patients needed to treat to prevent one ischaemic event compared with the 133 needed to cause one TIMI major bleed and 1000 to cause one fatal or intracranial bleeding event. Dual antiplatelet therapy for a year or more after acute myocardial infarction might, therefore, be considered in selected patients without previous stroke or anticoagulant therapy who are at greatest risk of recurrent ischaemic events. Our data showed that those aged ≥65, with diabetes, chronic renal disease, or previous acute myocardial infarction had a particularly high age-sex adjusted risk of recurrent acute myocardial infarction, stroke, or death, ranging from 20.8% to 32.3%. Our data also showed that across all CALIBER populations, however, adherence to secondary prevention drugs declined steeply during follow-up, despite high prescription rates after discharge after the index event. Non-adherence to secondary prevention drugs after acute myocardial infarction has been widely reported^34^ ^35^ ^36^ and for most patients must represent a more important target for improving outcomes than extended dual antiplatelet therapy.

### Implications of findings

Our findings have implications for guideline groups and clinicians. Guideline groups should seek to determine the representativeness of participants in clinical trials and the potential public health impact in terms of numbers of events prevented or harms caused in target and real world populations. The electronic health record resource we describe is scalable and offers considerable potential for answering these questions, thereby placing trial results in a clinical context. The clinical implications of our study are also relevant in addressing the applicability of trial findings in the consulting room. This is a question that commonly confronts clinicians, and the methods we present provide a novel means of addressing it. Our study also identifies a need for careful clinical supervision of patients who survive a year or more after acute myocardial infarction. It was an important observation that use of secondary prevention drugs declined progressively while ischaemic event rates were in steep ascent. This applied to all drug classes and represents a missed opportunity to protect these high risk patients against ischaemic events.

### Strengths and weaknesses

A particular strength of this study lies in its use of CALIBER’s linked electronic health records to assemble large target and real world populations for comparison with participants in a major randomised trial. In sourcing our real world patients from linked electronic health records, without exclusions and regardless of what treatment they received, we avoided confounding by indication and minimised biases in recruitment and follow-up. Data quality has been verified in studies that have established the diagnostic and prognostic validity of a range of risk factors.^16^ ^17^ ^18^ ^19^ ^20^ We have further assured highly complete event ascertainment, the diagnostic validity of primary care and hospital admission records for acute myocardial infarction showing positive predictive values of 92.2% and 91.5%, respectively, compared with a “ideal standard” diagnosis based on troponin values, findings on electrocardiography, and cardiological assessment in the MINAP disease registry.^12^ Moreover, there was no loss to follow-up in our CALIBER population in contrast with PEGASUS-TIMI-54. Other investigators have reported on event ascertainment within linked dual antiplatelet therapy. A comparative analysis within the West of Scotland Coronary Prevention Study (WOSCOPS) population concluded that it can be “as effective as reporting based on direct contact with patients.”^37^ Twenty year follow-up for cardiovascular events recorded in electronic health records has now been reported.^38^

It was a limitation of our study that we were unable to compare severity of coronary disease between the two populations as this information is unavailable in CALIBER. This is unlikely to have affected our conclusions, however, as there was no apparent heterogeneity in the efficacy of ticagrelor with respect to the severity of coronary disease in the trial. It was a further limitation of our study that we were able to provide a precise match for only two of the TIMI major bleeding criteria,^39^ the third criterion (≥50 g/L fall in haemoglobin concentration) required proxies: admission to hospital for bleeding with length of stay of seven days or more or need for blood transfusion. PEGASUS-TIMI-54’s other harm endpoint—fatal or intracranial bleeding—was matched in our CALIBER population. Estimates of the harms and benefits of extended dual antiplatelet therapy are based on relative risks calculated in PEGASUS-TIMI-54. The estimates are likely robust for the target group matched for trial selection criteria but require more cautious interpretation in the high risk and real world populations.

### Conclusions

In summary, this novel comparative analysis has shown that patients who survive a year or more after acute myocardial infarction remain at substantial risk of further cardiovascular events. The PEGASUS-TIMI-54 trial was one of the first to inform management of this understudied group. It recruited high risk patients, but our study has shown that real world patients who survive a year or more are at yet greater risk, amplifying the potential of dual antiplatelet therapy to improve prognosis. This potential must be weighed against the risk of bleeding and is likely to be greatest in high risk subgroups without previous stroke or recent anticoagulation therapy.

**What is already known on this topic**

Lifelong combinations of drug treatments are recommended for secondary prevention after acute myocardial infarction
The recent PEGASUS-TIMI-54 trial showed that prolonged dual antiplatelet therapy (addition of ticagrelor to low dose aspirin) in patients who had already survived to the first anniversary of their heart attack reduced the risk of ischaemic events but increased the risk of major bleeding

**What this study adds**

About a quarter of unselected patients a year or more after acute myocardial infarction might meet trial inclusion and exclusion criteria for treatment
Risk of recurrent acute myocardial infarction, stroke, or cardiovascular death in the next three years in these “target” patients was nearly 19%, twice the risk seen in the trial; this risk was similar (17%) in unselected (“real world”) patients
The potential for dual antiplatelet therapy to deliver clinical benefit a year or more after acute myocardial infarction is likely to be greatest in high risk subgroups without previous stroke or recent anticoagulation

## Web Extra

Extra material supplied by the author

## Notes

Contributors: AT contributed to the writing of the manuscript and the interpretation of results. SD prepared the data. ER and SCC supervised the data analysis, assisted by MP-R and DS. ADS, LP, CE, and HH critically reviewed and commented on the manuscript. CE, an employee of the sponsor, co-authored the paper based on contributions she made to interpreting the data and editing the report. All authors reviewed and approved the final version of the manuscript. ER and SD are guarantors.

Ethical approval: The study was part of the CALIBER programme, approved by the MINAP Academic Group and by the CPRD independent scientific advisory committee (protocol 13_163R).

Data sharing: No additional data available. The linked data used for this research are available for scrutiny at the Farr Institute London but cannot be released because of the licensing conditions to which we are obliged to adhere.

Transparency: The lead author, AT, affirms that the manuscript is an honest, accurate, and transparent account of the study being reported; that no important aspects of the study have been omitted; and that any discrepancies from the study as planned have been explained.
