# Supplementary material for: Prolonged dual antiplatelet therapy in stable coronary disease: comparative observational study of benefits and harms in unselected versus trial populations
Source: BMJ. 2016 Jun 22;353:i3163. doi: 10.1136/bmj.i3163 (PMC4916922; doi:10.1136/bmj.i3163)
Supplement: Supplementary file 1 — Appendix 1: Supplementary material and supplementary tables A-E [file tima031610.ww1_default.pdf]

Appendix 1 Bleeding Definition and Classifications and supplementary tables [posted as supplied by author]

Bleeding Definition and Classifications

1. **Hospitalized bleeding**: bleeding ICD-10 code (table ST2) recorded in HES first admission
2. **Fatal bleeding**: bleeding documented in icd10_death and a HES admission with bleeding ICD-10 code (table T) that occurred 7 days prior to death.
3. **Intracranial bleeding:** bleeding in the brain (ICD10 code I60, I61, I62) recorded in HES first admission
4. **Bleeding requiring transfusion:** a bleeding event that led to transfusion documented in OPCS-4 or transfusion documented in the Standard clinical terminology system used in General Practice in the United Kingdom (READ codes) within 30 days.

   READ codes for transfusion:

7L14.00	Other blood transfusion	11452
7L14000	Intra-arterial blood transfusion	70681
7L14100	Intravenous blood transfusion of packed cells	43127
7L14300	Intravenous blood transfusion NEC	22458
7L14311	Blood transfusion	796
7L14y00	Other specified other blood transfusion	64115
7L14z00	Other blood transfusion NOS	45074
TAy0.00	Mismatched blood transfused	65166
TB1y000	Blood transfusion with complication, without blame	63774
ZV58200	[V]Blood transfusion, without reported diagnosis	22153

   OPCS codes for transfusion:

X33	Other blood transfusion
X331	Intra-arterial blood transfusion
X332	Intravenous blood transfusion of packed cells
X333	Intravenous blood transfusion of platelets
X338	Other specified other blood transfusion
X339	Unspecified other blood transfusion

5. **Definition of severe bleeds:** bleeding required transfusion (definition 4) or hospitalized bleeding with length of stay ≥ 7 days

Table A Bleeding definitions based on ICD10 codes

Condition*	ICD 10 codes	
Bleeding	Bleeding in the brain	
	I60 (I600–I609)	Subarachnoid haemorrhage
	I61 (I610–I619)	Intracerebral haemorrhage
	I62 (I620, I621, I 629)	Other non-traumatic intracranial haemorrhage
	Gastrointestinal bleeding	
	K25·0	Gastric ulcer; acute with haemorrhage
	K25·2	Gastric ulcer; acute with both haemorrhage and perforation
	K25·4	Gastric ulcer; chronic or unspecified with haemorrhage
	K25·6	Gastric ulcer; chronic or unspecified with both haemorrhage and perforation
	K26·0	Duodenal ulcer; acute with haemorrhage
	K26·2	Duodenal ulcer; acute with both haemorrhage and perforation
	K26·4	Duodenal ulcer; chronic or unspecified with haemorrhage
	K26·6	Duodenal ulcer; chronic or unspecified with both haemorrhage and perforation
	K27·0	Peptic ulcer, site unspecified; acute with haemorrhage
	K27·2	Peptic ulcer, site unspecified; acute with both haemorrhage and perforation
	K27·4	Peptic ulcer, site unspecified; chronic or unspecified with haemorrhage
	K27·6	Peptic ulcer, site unspecified; chronic or unspecified with both haemorrhage and perforation
	K28·0	Gastrojejunal ulcer; acute with haemorrhage
	K28·2	Gastrojejunal ulcer; acute with both haemorrhage and perforation
	K28·4	Gastrojejunal ulcer; chronic or unspecified with haemorrhage
	K28·6	Gastrojejunal ulcer; chronic or unspecified with both haemorrhage and perforation
	K29·0	Acute haemorrhagic gastritis
	K62·5	Haemorrhage of anus and rectum
	K92·0	Haematemesis
	K92·1	Melaena
	K92·2	Gastrointestinal haemorrhage, unspecified
	Other location	
	H35·6	Retinal haemorrhage
	H43·1	Vitreous haemorrhage
	H45·0	Vitreous haemorrhage in diseases classified elsewhere
	R04·1	Haemorrhage from throat
	R04·8	Haemorrhage from other sites in respiratory passages
	R04·9	Haemorrhage from respiratory passages, unspecified

Table B Medication codes

	England (BNF codes)
Clopidogrel	BNF 2.9, substance clopidogrel
Prasugrel	BNF 2.9, substance prasugrel
Aspirin	BNF 2.9, substance aspirin
Statins	BNF 2.12
Beta-blockers	BNF 2.4
Calcium channel blockers	BNF 2.6.2
ACEI or ARBs	BNF 2.5.5.1<br>BNF 2.5.5.2
Diabetes medication	BNF 6.1.1<br>BNF 6.1.2

ACEI, angiotensin-converting enzyme inhibitor; ATC, Anatomical Therapeutic Chemical classification system; BNF, British National Formulary classification codes; ARB, angiotensin II receptor blocker

Table C: Observed cumulative rate % of drug treatment (with 95% CI) in the UK post-MI patients populations defined in the study.

	All post-MI survivors ‘Real world’	Met trial inclusion criteria ‘High risk’	Met trial inclusion & exclusion criteria ‘Target ’
Statin			
1-year	80.9 (80.0-81.9)	78.9 (77.8-80.0)	73.8 (71.7-76.0)
2-year	72.2 (71.0-73.3)	69.5 (68.1-71.0)	65.2 (62.7-67.8)
3-year	62.4 (60.9-64.0)	59.9 (58.1-61.8)	55.5 (52.4-58.7)
Beta-blockers			
1-year	65.1 (63.9-66.2)	62.2 (60.8-63.5)	59.1 (56.7,61.5)
2-year	57.6 (56.3-58.9)	54.6 (53.1-56.1)	51.6 (49.1-54.4)
3-year	50.4 (48.9-51.9)	47.1 (45.3-48.9)	44.5 (41.5-47.7)
ACEI/ARB			
1-year	71.2 (70.1-72.2)	69.1 (67.8-70.4)	66.7 (64.5-69.1)
2-year	59.3 (58.0-60.6)	56.2 (54.6-57.7)	54.7 (52.1-57.5)
3-year	45.2 (43.7-46.8)	41.9 (40.1-43.7)	41.3 (38.2-44.6)
Antiplatelet (aspirin)			
1-year	74.9 (73.9-76.0)	73.1 (71.8-74.3)	74.9 (73.9-76.0)
2-year	65.7 (64.5-66.9)	63.2 (61.7-64.7)	65.7 (64.5-66.9)
3-year	55.5 (54.0-57.1)	53.1 (51.3-55.0)	55.5 (54.0-57.1)
Antiplatelet (ADP receptor inhibitors)			
1-year	17.6 (16.7-18.5)	18.2 (17.1-19.3)	N/A
2-year	11.3 (10.5-12.2)	11.9 (10.9-12.9)	
3-year	8.0 (7.2-8.9)	8.3 (7.4-9.4)	

Table D. Observed cumulative event rate % of clinical outcome and number of harm caused applying trial results to the UK post-MI patients populations defined in the study (with 95% CI).

	Population based observational data			Trial
	All post-MI survivors 'Real world' n=7238	Met trial inclusion criteria 'High risk' n=5279	Met trial inclusion & exclusion criteria 'Target ' n=1676	Placebo arm in PEGASUS-TIMI-54 trial N=6996
**Fatal or intracranial bleeding**				
3-year cumulative risk (%)	1.1 (0.8,1.5)	1.4 (1.0,2.0)	1.5 (0.8,2.7)	0.6
Number excess harms per year per 10,000 patients treated applying risk increase in PEGASUS-TIMI-54 trial[1]	8 (6-10)	10 (7-14)	10 (6-18)	

[1]Based on applying the trial on-treatment relative risks of the ticagrelor 60 mg vs. placebo comparison: 1.20 (fatal or intracranial bleeding) to the CALIBER populations.

Table E. One year and 3 year observed Kaplan-Meier risks (%, 95% CI) of MI stroke of CVD death according to clopidogrel use post-MI patients populations defined in the study, by prescribed clopidogrel at one year past stable date status (with 95% CI).

	Population based observational data			PEGASUS-TIMI-54 Trial
	All post-MI survivors 'Real world' (n=7238)	Met trial inclusion criteria 'High risk' (n=5279)	Met trial inclusion & exclusion criteria 'Target ' (n=1676)	Placebo arm
	**Censored within a year after study entry**			
**a) Clopidogrel not prescribed at entry**				
Patients (n)	1211	981	609	N/A
MI/stroke/ CVD deaths (n)	207	199	118	
1-year Kaplan-Meier cumulative risk (%)	59.8 (38.3,82.1)	63.7 (43.3,83.6)	55.6 (32.7,81)	
**b) Clopidogrel prescribed at entry**				
Patients (n)	1310	977	0	N/A
MI/stroke/CVD deaths (n)	243	222	0	
1-year Kaplan-Meier cumulative risk (%)	53.4 (43.9,63.5)	69.8 (54.1,84.1)	N/A	
	**1 year after study entry (2 years past index AMI)**			
**a) Clopidogrel not prescribed**				
Patients (n)	2809	1999	1047	n=7067
MI/stroke/ CVD deaths (n)	187	116	81	
3-year Kaplan-Meier cumulative risk (%)	9.8 (8.4,11.5)	12.4 (10.5,14.5)	11.5 (9.1,14.5)	9.04
**b) Clopidogrel prescribed**				
Patients (n)	1908	1322	20	N/A
MI/stroke/CVD deaths (n)	161	144	3	
3-year Kaplan-Meier cumulative risk (%)	12.1 (10.3,14.2)	15.6 (13.2,18.3)	18.9 (6.3,49.1)	
