# Supplementary material for: Prolonged dual antiplatelet therapy in stable coronary disease: comparative observational study of benefits and harms in unselected versus trial populations
Source: BMJ. 2016 Jun 22;353:i3163. doi: 10.1136/bmj.i3163 (PMC4916922; doi:10.1136/bmj.i3163)

Appendix 2: Supplementary figure [posted as supplied by author]

Fig A. Kaplan-Meier risks for all-cause mortality in CALIBER post-MI survivor populations (all, high-risk and target). The Kaplan-Meier all-cause mortality of the PEGASUS-TIMI-54 placebo group was 5.16%

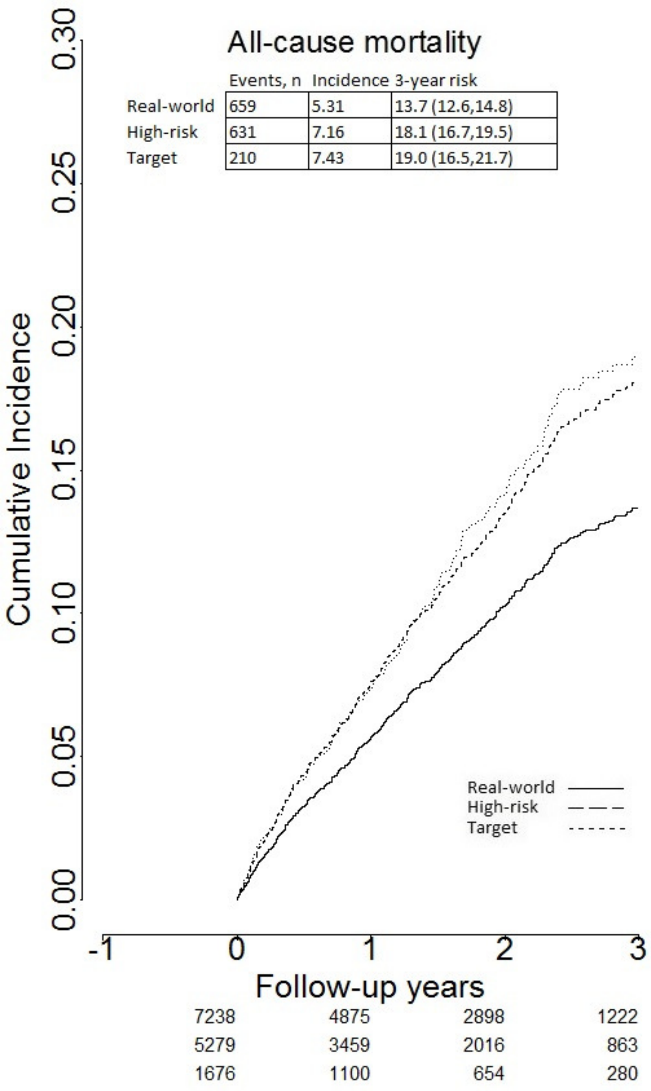

Supplement: Supplementary file 2 — Appendix 2: Supplementary figure A [file tima031610.ww2_default.pdf]
